# Movement Velocity and Fluidity Improve after Armeo®Spring Rehabilitation in Children Affected by Acquired and Congenital Brain Diseases: An Observational Study

**DOI:** 10.1155/2018/1537170

**Published:** 2018-11-18

**Authors:** Emilia Biffi, Cristina Maghini, Beatrice Cairo, Elena Beretta, Elisabetta Peri, Daniele Altomonte, Davide Mazzoli, Meris Giacobbi, Paolo Prati, Andrea Merlo, Sandra Strazzer

**Affiliations:** ^1^Scientific Institute IRCCS Eugenio Medea, Bosisio Parini, Lecco, Italy; ^2^Gait & Motion Analysis Laboratory, Sol et Salus Hospital, Rimini, Italy; ^3^Sol et Salus Hospital, Rimini, Italy

## Abstract

**Background:**

Children with cerebral palsy (CP) and acquired brain injury (ABI) often exhibit upper limb impairment, with repercussions in their daily activities. Robotic rehabilitation may promote their functional recovery, but evidence of its effectiveness is often based on qualitative functional scales. The primary aim of the present work was to assess movement precision, velocity, and smoothness using numerical indices from the endpoint trajectory of Armeo®Spring. Secondly, an investigation of the effectiveness of robotic rehabilitation in CP and ABI children was performed.

**Methods:**

Upper limb functional changes were evaluated in children with CP (N=21) or ABI (N=22) treated with Armeo®Spring (20 45-minute sessions over 4 weeks) using clinical scales and numerical indices computed from the exoskeleton trajectory.

**Results:**

Functional scales (i.e., QUEST and Melbourne) were sensitive to changes produced by the treatment for the whole study group and for the two etiology-based subgroups (improvements above Minimal Clinically Importance Difference). Significant improvement was also observed in terms of velocity, fluidity, and precision of the movement through the numerical indices of kinematic performance. Differences in the temporal evolution of the motor outcome were highlighted between the ABI and CP subgroups, pointing toward adopting different rehabilitative protocols in these two populations.

**Conclusions:**

Robot-assisted upper limb rehabilitation seems to be a promising tool to promote and assess rehabilitation in children affected by acquired and congenital brain diseases.

## 1. Introduction

Upper limb impairments may cause important limitations in daily life activities of affected patients. When this condition occurs in childhood, it can restrict the typical development of subjects with severe consequences on the ability to reach, grasp, and manipulate objects, with significant impacts on the ability of children to perform daily activities and develop the skills required to participate in activities at home, school, and community.

Acquired Brain Injuries (ABI) are nonprogressive, nonhereditary brain injuries that occur after birth, as a consequence of different events, such as trauma, hypoxia, stroke, and infection. The resulting long-term disability can include deficiencies in cognitive, behavioral, metabolic, motor, perceptual motor, and/or sensory brain functions and often include upper limb limitations [1].

Cerebral palsy (CP) is a neurodevelopmental condition beginning in early childhood, featured by various abnormal patterns of movement and posture due to defective coordination of movement and/or regulation of muscle tone [2]. Children with CP are often characterized by problems with arm and hand functionality [3].

Several treatments have been tested on children with upper limb impairments; among them, constraint-induced movement therapy and bimanual intensive therapy are recognized as the most used [4, 5]. These treatments have been backed up by robot-aided therapy that can support repetitive and high-intensity training tasks in motivating environment when combined with virtual reality exergames [6–9].

Upper limb robot-aided therapy has shown good functional improvements in terms of coordination and fluency in the pediatric population, using both end-effector robots [8, 10–14] and passive exoskeletons [15, 16].

Robot-aided therapy has some additional advantages that can contribute to the understanding of the effectiveness of these devices in motor learning and recovery [17, 18]. It has indeed the ability of measuring patients' movement kinematics and forces during treatments instead of relying exclusively on qualitative observation. Moreover, it has higher resolution as well as better interrater and intrarater reliability with respect to clinical scales. It has been shown that robot-based measurements can be correlated with clinical scales assessing adults' [17, 19–21] and children's [15, 22] abilities: these data can support clinicians in the assessment of a patient's progress and capabilities. Recently, Merlo and coworkers developed a tool to exploit the built-in technology of the exoskeleton Armeo®Spring to extract indexes of accuracy, velocity, and smoothness in the evaluation of upper limb function and obtained normative data in healthy adults [23]. The validity of this tool was then demonstrated in adult poststroke patients, using the indices extracted to distinguish between poststroke patients and healthy subjects [24]. The authors concluded that the tool could be used to support the clinical evaluations of patients. However, up to now the indices proposed have never been used in pediatric population with assessment purpose.

The primary aim of the present study was to use numerical indices assessing movement accuracy, velocity, and smoothness computed from a 3D endpoint trajectory, as described in [23]. These indexes were used to support the evaluation of the functional changes occurring in children affected by congenital or acquired brain damage after a training with the Armeo®Spring device. Furthermore, an additional goal was the retrospective evaluation of the effectiveness of robot-aided rehabilitation used at the Scientific Institute Eugenio Medea to improve the upper limb functionality of children. The assessment was supported by pre/post evaluations by means of both functional scales and quantitative measurements derived from the data, as automatically recorded by the exoskeleton.

## 2. Methods

### 2.1. Participants

In this retrospective study, we included patients that took part in a rehabilitation treatment with the Armeo®Spring device at the Neurophysiatric Area of the Scientific Institute Eugenio Medea, Bosisio Parini, Italy. The inclusion criteria usually followed by the clinicians of the institute were age between 5 and 18; the ability to handle objects in daily life within levels I, II, and III, according to the Manual Ability Classification System (MACS); and the ability to understand and follow test instructions. Conversely, the exclusion criteria were severe muscle spasticity and/or contracture, a diagnosis of severe learning disabilities or behavioral problems and visual or hearing difficulties that would impact on function and participation.

According to the inclusion criteria, 43 patients were considered for the current study, 22 children with ABI and 21 affected by CP, whose demographic characteristics are summarized in Table 1.

The protocol was approved by the central ethical committee of Scientific Institute Eugenio Medea on the 12th of April 2018 and conducted in accordance with the Declaration of Helsinki. The ethical committee stated that the informed consent was not required for this retrospective observational study. The study has been registered in clinicaltrial.gov (registration number NCT03552614). The clinical and instrumental data used to support the findings of this study are available from the corresponding author upon request.

### 2.2. Armeo Training

Armeo®Spring is a passive exoskeleton with five degrees of freedom which guarantees passive arm weight support and guidance with springs. The device is coupled to virtual exergames that provide visual feedback during therapy. The features of the arm support and of the exergames can be adapted to the patient's individual morphology and residual ability.

**Figure 1:**
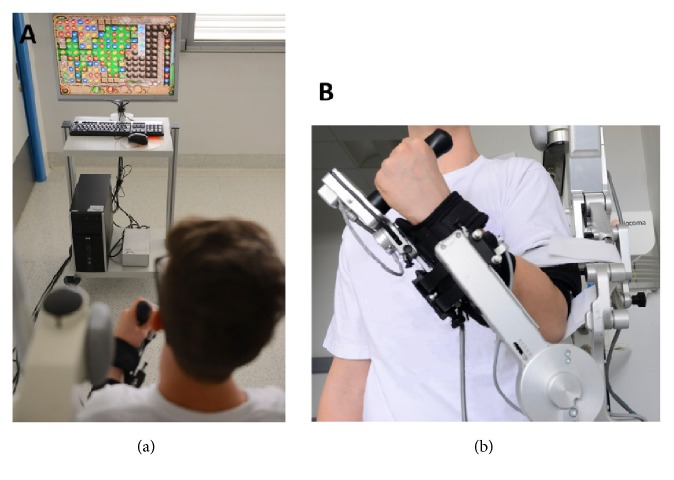
Experimental setup. (a) Patient performing an exergame with Armeo®Spring. (b) The Armeo®Spring worn by the patient on his left arm.

Patients performed 45-minute treatment sessions 5 times a week for 4 weeks with Armeo®Spring according to the standard protocol of the IRCCS E. Medea (Figure 1). In each session, patients repetitively played exergames that simulate meaningful tasks targeting different upper arm joints and regions. At the beginning of the treatment (T0), during the fifteenth session (Tm) and at the end of the treatment (T1), patients executed the Armeo “Vertical Capture” exergame, which assesses patients' functional level and requires reaching a target (a ladybird) with a cursor controlled by the endpoint position of the exoskeleton in the 2-dimensional space. This task involves elbow flex-extension and shoulder flex-extension and abd-adduction (see [23] for more details). The “Vertical Capture” exergame was not executed during the other training sessions.

Physiotherapists oversaw each session and adjusted exercises, weight compensation, and maximal active workspace according to the progress of each patient. Furthermore, they increased the difficulty level and number of repetitions of the games and introduced more difficult games into the training system according to the patients' abilities. In contrast, all the Armeo parameters were maintained constant during T0, Tm, and T1 evaluations.

In addition to the robotic training, patients underwent 45-minute treatment sessions 5 times a week for 4 weeks of physiotherapy that focused on gross and fine motor ability to promote independence in daily activities, and it was customized on patients' need.

### 2.3. Outcome Measures: Functional Scales and Instrumental Data

Patients were evaluated before (T0) and after (T1) the intervention with the Quality of Upper Extremities Skills Test (QUEST) and the Melbourne Assessment of Unilateral Upper Limb Function [5, 16, 25], according to the evaluation protocol defined at the IRCCS E. Medea.

The QUEST is an internationally validated scale that measures dissociated movement, grasp, weight-bearing, and protective extension abilities in children with upper extremity movement disorders. The total score is the average of these four domain scores, with higher scores representing a better quality of movement.

The Melbourne Assessment is a test that scores the quality of unilateral upper-limb motor function based on items involving reach, grasp, release, and manipulation in neurologically impaired children.

Furthermore, kinematic data measured by the Armeo potentiometers during the execution of the “Vertical Capture” exergame and the endpoint trajectory were acquired and stored by the system. Indices of accuracy, velocity, and smoothness were then computed from the 3D endpoint trajectory, with the Matlab-based tool described in [23], at T0, Tm, and T1. The indices extracted were

(i) the hand path ratio (HPR), i.e., the ratio between the pathway of the end effector and the shortest trajectory between the initial and final positions; this parameter is equal to 100% if straight movements are performed while it increases when curved trajectories occur;

(ii) the horizontal and vertical overshooting of the movement (in cm) with respect to the target (horOS, verOS), a measure of the deviation from the target point (i.e., precision);

(iii) the mean and the maximum velocity of the 3D endpoint trajectory (cm/s);

(iv) the number of peaks of the velocity profile (NvelPeaks);

(v) the normalized jerk (NormJerk), computed as the differentiation of the 3D endpoint trajectory. Its value increases for irregular trajectories.

 HPR, horOS, and verOS were considered measures of task precision, while NvelPeaks and NormJerk were used to evaluate trajectory smoothness.

### 2.4. Statistical Analysis

The Shapiro-Wilk test was performed to check the normality of data. Since not all the measures were normal, a nonparametric statistical analysis was carried out. Specifically, the Wilcoxon test was used to compare functional evaluations before and after the treatment for the whole group as well as for the two subgroups (acquired and congenital brain injury). Moreover, the Friedman test and the post hoc Bonferroni-corrected Wilcoxon test were applied on instrumental indices to evaluate changes of these data. The Mann–Whitney U test was run to compare the two subgroups at the beginning of the treatment. Finally, a linear correlation analysis among the functional data and the indices extracted by the Matlab-based tool was carried on at T0 and T1 (Spearman's rho). The significance level was set at 5%. These analyses were performed with SPSS.

## 3. Results

Table 2 shows the results relative to the functional evaluations for the whole group and for the two subgroups across time: improvements both in the QUEST and Melbourne scales can be observed after treatment for the entire study group.

The Mann–Whitney U test run on the values of the functional scales found no significant differences between the two clinical subgroups at the beginning and at the end of the treatment, even if, at both time points, median values were worse for the ABI group with respect to CP. Both groups showed improvements between T0 and T1 in terms of QUEST and Melbourne scales, with a large effect size, except for the Melbourne scale in the ABI group (medium effect size) [26].

Table 3 describes the results about the indices extracted with the Matlab-based tool, realized by Merlo and collaborators, on the whole study group. In particular, it can be observed that there are significant improvements between T0 and Tm in all parameters, except for verOS; this improvement is also maintained at T1. The mean velocity and NvelPeaks show further improvements between Tm and T1.

When the Mann–Whitney U test was performed on these parameters, the only difference found between subgroups at T0 was in terms of NormJerk, which was significantly higher in patients affected by ABI (p=0.014). As presented in Table 4, results in patients affected by ABI were similar to those of the whole group, although improvements between T0 and Tm, maintained at T1, remained only for the mean, the maximum velocity, and the number of peaks of velocity, and the normalized jerk improved only between T0 and T1. On the other hand, significant improvements were observed on the velocity parameters in the CP group: specifically, the mean and maximum velocity increased between Tm and T1 while NvelPeaks improved between T0 and Tm and was maintained at T1. No differences between ABI and CP were detected after the treatment.

Finally, the correlation analysis at T0 highlighted good correlation between functional evaluations and instrumental data. Specifically, the QUEST was moderately correlated with the HPR (rho=-0.67, p<0.001), the horOS (rho=-0.47, p=0.005), the NvelPeaks (rho=-0.56, p<0.001), and the NormJerk (rho=-0.67, p<0.001). Similarly, the score of the Melbourne scale was moderately correlated with the same variables: the HPR (rho=-0.63, p<0.001), the horOS (rho=-0.47, p=0.004), the NvelPeaks (rho=-0.60, p<0.001), and the NormJerk (rho=-0.68, p=<0.001). The correlations were preserved and often even strengthened over time. At T1, the QUEST was indeed correlated with the HPR (rho=-0.65, p<0.001), the horOS (rho=-0.70, p<0.001), the NvelPeaks (rho=-0.71, p<0.001), and the NormJerk (rho=-0.64, p=<0.001). Similarly, the score of the Melbourne scale was moderately correlated with the same variables: the HPR (rho=-0.52, p=0.003), the horOS (rho=-0.61, p<0.001), the NvelPeaks (rho=-0.60, p=<0.001), and the NormJerk (rho=-0.58, p=<0.001).

## 4. Discussion

Children with central nervous system lesions may present important limitations in the use of upper extremities which cause deficits in activities of daily living. In recent years, robotic rehabilitation has become a new tool for upper limb functional recovery in patients with CP and ABI [8, 10–16].

In this study, upper limb functional changes were evaluated in children with CP or ABI treated with Armeo®Spring. Patients were evaluated using clinical scales and numerical indices extracted from the exoskeleton before and after treatment. Two were the main aims of this work: to verify if numerical indices computed as described in [23] can assess movement accuracy, velocity, and smoothness in children affected by congenital or acquired brain damage and to retrospectively evaluate the effectiveness of robot-aided rehabilitation with Armeo®Spring used at the Scientific Institute Eugenio Medea to improve the upper limb functionality of children.

Concerning the first goal, the Melbourne scale and the QUEST showed significant correlations with indices describing movement straightness, task precision, and trajectory smoothness. The correlation between functional scales and the indices extracted attests the validity of the latter in evaluating the motor outcome in a pediatric population affected by acquired or congenital brain injury. This is in line with the literature: a recent review [27] highlights that kinematic parameters recorded by robots have been proposed as indicators of motor performance.

So far, the evaluation of the motor recovery is mainly based on clinical scales that are often administered before and after a therapeutic intervention. In contrast to clinical outcome measures, kinematic measures obtained by sensors on robots can be easily analyzed after each training session, providing new assessment measures with improved objectivity, repeatability, precision, and ease of application. The possibility of recording quantitative measures every session or every task allows to monitor the course of treatment and provide the therapist with real-time, objective measures of patient motor capabilities. This can help to follow patient progress, to evaluate the effectiveness of different interventions, or to adapt to specific patients' motor recovery trend [27].

It is worth noticing that, in this work, the indices assessing movement accuracy, velocity, and smoothness computed from a 3D endpoint trajectory, as described in [23] and used to evaluate differences between healthy and poststroke adult patients [24] were used to evaluate the effects of a robot-aided rehabilitation in the pediatric population for the first time. Therefore, these numerical indices can be included in the clinical practice to support the evaluation of functional changes occurring in children affected by congenital or acquired brain damage after a training with the Armeo®Spring device.

Concerning the second goal, functional scales showed significant improvements in the whole study group and in the two etiology-based subgroups (CP and ABI), suggesting the clinical effectiveness of this rehabilitative tool, associated with physiotherapy, and a lack of significant differences between subgroups. The values of these improvements were above the Minimal Clinically Importance Difference (MCID) for the QUEST (MCID=3.5) but not for the Melbourne scale (MCID=4.3), estimating the MCID with the standard error of measurement (SEM) as suggested by [28]. However, Revicki and collaborators suggest that minimal differences may be bigger than clinically meaningful differences: a two-point improvement in the Melbourne scale was indeed defined satisfactory by clinicians involved in this study.

The positive results obtained using the clinical scales were confirmed by the numerical indices, which showed an increase of the movement velocity as well as of its smoothness and task precision.

The improvement for most parameters was significant between T0 and Tm and maintained at T1. Furthermore, mean velocity and number of peaks of velocity showed further significant improvement in the second part of the treatment (Tm-T1), highlighting the importance of training dose in the recovery of pediatric patients.

The concurrent improvements of functional scales and numerical indices support that the changes observed cannot be only ascribed to learning effect. Furthermore it is noteworthy that the indices were extracted from an exergame not specifically trained during the protocol.

Numerical indices showed stronger improvements during treatment in patients affected by ABI with respect to CP: ABI patients had less residual functionality at T0, as shown by the functional scales, and this difference at baseline, even if not significant, might have influenced the rehabilitation outcome since milder patients can experience a plateau in their performance. Further, recovery is very different in CP and ABI even if they may show a similar neurological impairment.

Differences in the temporal evolution of the motor outcome were highlighted between ABI and CP subgroups. Indeed, ABI and CP had comparable functional ability at baseline and had no differences at the end of the training; but, progress in the ABI group was significant in the first part of the training, while the CP group improved mostly in the second part of the rehabilitation. This may point toward the importance of adopting different rehabilitative protocols to treat upper limb impairments in these two pediatric populations.

The present study investigates the effectiveness of Armeo®Spring-based rehabilitation, coupled to physiotherapy, on a population which few other studies on the same topic have analyzed. Few manuscripts have already reported functional improvements in CP children after robot-aided rehabilitation with Armeo®Spring; the improvement reported here in terms of Melbourne scale was as significant as in [15, 16], even though the patients had lower scores at baseline and the variation between baseline and final evaluation was smaller as well. In contrast, they showed a significant improvement in the QUEST scale that was not reported in [16]. Furthermore, the data shown in the present work were collected on a wider sample size than previous ones [15, 16] and, to our knowledge, are the first indication of the effectiveness of Armeo®Spring rehabilitation in addition to physiotherapy on children suffering from ABI.

Progress we obtained with a passive exoskeleton seems to be in line with those conveyed by end-effectors robots. Indeed, Frascarelli and collaborators found statistically significant changes in motor performance from admission to discharge, with moderate to large effect sizes on the Melbourne Assessment in a mix population (10 ABI + 2 CP) [8]. Furthermore, Ladenheim and coworkers observed improvements in motor capacity as measured by the Fugl Meyer Assessment of Motor Function in a group of 31 children, including both ABI and CP [13].

The high variability within the group of study and the absence of a control group that performs only physiotherapy and of a follow-up evaluation limit the generalization of our results. Furthermore, data of healthy subjects would constitute normative values on the pediatric population. However, this paper underlines the importance of a treatment coupling exergames performed with a passive exoskeleton to physiotherapy in children with cerebral impairment and how the treatment produces a comprehensive improvement of the functionality of the limbs both quantitatively and qualitatively in the entire study group and in both subgroups. Future studies should tackle the evaluation of treatment effects on children brain activity using neurophysiology and neuroimaging techniques. Recent studies have indeed evidenced the correlation between functional scales and brain lesions [29, 30].

## 5. Conclusions

The study is relevant to both clinical and bioengineering fields. Indeed, the instrumental indices extracted from robotic built-in sensors to assess quality of movement in children affected by congenital and acquired brain damage have proven to be effective and promising tools to integrate clinical evaluation of upper limb functionality. Furthermore, robot-assisted therapy in addition to physiotherapy conveys upper limb functional improvements in children affected by cerebral palsy and acquired brain injury.

## Figures and Tables

**Table 1:** Demographic features of the patients included into the study.

	**ABI+CP (N=43)**	**ABI (N=21)**	**CP (N=22)**
**Age at T0 (years)**	11.1(5.8)	14.4(9.2)	10.8(4.2)

**Age at trauma (years) ** **∗**	10.0(8.9)	10.0(8.9)	-

**Gender (M/F)**	24/19	8/13	16/6

**Etiology (TBI/T/HE/CP/OP)**	5/8/8/21/1	5/8/8/0/0	0/0/0/21/1

**Distance of the treatment from the brain damage (years)** **∗** **∗**	7(8.1)	2.6(3.6)	10.8(4.2)

**MACS** **(I/II/III/IV/V)**	3/13/22/4/1	0/5/12/4/0	3/8/10/0/1

**Trained arm (R/L)**	25/18	11/10	14/8

Mean (standard deviation) is reported. M: male, F: female, TBI: traumatic brain injury, T: tumor, HE: hemorrhagic, CP: cerebral palsy, OP: obstetric paralysis. R: right, L: left. *∗* Valid only for ABI. *∗∗*These data correspond to the age for PCI and OP.

**Table 2:** Results about functional evaluations (QUEST and Melbourne scales).

		**T0** **Median (IQR)**	**T1** **Median (IQR)**	**p Wilcoxon T0 vs T1**	**Effect size**
**ABI+CP (N=35)**	**Quest TOT**	70.3 (22.7)	74.3 (22.5)	<0.001	0.9
	**Melbourne **%	73 (19.5)	75 (17)	<0.001	0.8

**ABI (N=17)**	**Quest TOT**	60.4 (13.7)	67.6 (17.7)	0.002	0.8
	**Melbourne **%	65 (42)	65 (41)	0.016	0.6

**CP (N=18)**	**Quest TOT**	75.0 (15.3)	79.2 (11.9)	<0.001	0.9
	**Melbourne **%	79 (15.8)	80.5 (14.3)	0.005	0.9

Data obtained before (T0) and after (T1) intervention are reported. P values refer to the nonparametric paired Wilcoxon test. IQR: interquartile range.

**Table 3:** Results of instrumental measures on the entire study group.(CP+ABI, N=43).

		T0 Median (IQR)	Tm Median (IQR)	T1 Median (IQR)	p Friedman	p Wilcoxon Bonf-corrected		
						T0 vs Tm	T0 vs T1	Tm vs T1
**ABI+CP (N=43)**	**HPR (**%**)**	166.0 (52.9)	148.3 (30.0)	144.8 (47.3)	0.002	0.028	0.013	1.000
	**horOS [cm]**	1.8 (1.0)	1.3 (0.8)	1.1 (1.0)	0.011	0.034	0.010	1.000
	**verOS [cm]**	1.0 (0.3)	1.0 (0.5)	0.3 (0.8)	0.471			
	**Mean vel [cm/s]**	2.8 (1.5)	3.5 (1.3)	4.0 (1.0)	<0.001	<0.001	<0.001	0.005
	**Max vel [cm/s]**	6.8 (3.6)	9.8 (3.5)	10.5 (3.4)	<0.001	<0.001	<0.001	0.073
	**NvelPeaks**	2.0 (0.8)	1.8 (0.5)	1.5 (1.0)	<0.001	<0.001	<0.001	0.048
	**Norm jerk**	787 (1352)	298 (1019)	304 (394)	0.002	0.007	0.006	0.545

Parameters were extracted using the Matlab-based tool realized by Merlo and collaborators [23], before (T0), during (Tm) and after (T1) intervention. P values refer to the nonparametric paired Wilcoxon test. HPR: Hand Path Ratio; horOS: horizontal overshooting; verOS: vertical overshooting; Mean/Max vel: mean/maximum velocity; NvelPeak: number of velocity peaks; Norm jerk: normalized jerk.

**Table 4:** Results of instrumental measures on the subgroups.

		T0 Median (IQR)	Tm Median (IQR)	T1 Median (IQR)	p Friedman	p Wilcoxon Bonf-corrected		
						T0 vs Tm	T0 vs T1	Tm vs T1
**ABI (N=21)**	**HPR (**%**)**	169.8 (70.3)	155.5(28.8)	146.8 (49.8)	0.007	0.573	0.052	0.531
	**horOS [cm]**	2.3 (1.8)	1.3 (1.1)	1.3 (1.5)	0.039	0.193	0.197	1.000
	**verOS [cm]**	0.8 (0. 5)	0.8 (0.8)	1.0 (0.7)	0.709			
	**Mean vel [cm/s]**	3.0 (1.3)	3.8 (1.3)	4.0 (0.5)	0.001	0.001	0.004	0.505
	**Max vel [cm/s]**	8.8 (4.0)	11.0 (2.5)	10.6 (2.2)	0.001	0.001	0.004	1.000
	**NvelPeaks**	2.3 (1.0)	1.8 (0.7)	1.8 (1.4)	<0.001	0.008	<0.001	0.255
	**Norm jerk**	1391 (3119)	545 (1150)	437 (1106)	0.007	0.301	0.042	0.279

**CP (N=22)**	**HPR (**%**)**	152.0 (41.7)	143.3 25.3)	142.0 (38.3)	0.076			
	**horOS [cm]**	1.8 (1.4)	1.3 (1.3)	1.0 (0.8)	0.215			
	**verOS [cm]**	1.1 (1.2)	1.0 (1.0)	1.0 (0.3)	0.580			
	**Mean vel [cm/s]**	2.4 (1.0)	3.0 (1.3)	4.0 (1.6)	<0.001	0.055	0.001	0.008
	**Max vel [cm/s]**	6.3 (2.9)	8.0 (3.5)	10.0 (5.3)	<0.001	0.068	<0.001	0.014
	**NvelPeaks**	2.0 (0.5)	1.5 (0.5)	1.5 (0.5)	<0.001	0.013	0.008	0.174
	**Norm jerk**	369 (931)	212 (364)	284 (174)	0.143			

Parameters were extracted using the Matlab-based tool realized by Merlo and collaborators [23], before (T0), during (Tm), and after (T1) intervention. P values refer to the nonparametric paired Wilcoxon test. HPR: Hand Path Ratio; horOS: horizontal overshooting; verOS: vertical overshooting; Mean/Max vel: mean/maximum velocity; NvelPeak: number of velocity peaks; Norm jerk: normalized jerk.

## Data Availability

The data used to support the findings of this study are available from the corresponding author upon request.
